# Computer-Aided Virtual Saturation Mutagenesis Improves the Lignocellulose-Degrading Performance of an *Aspergillus niger* LPMO

**DOI:** 10.3390/foods15122178

**Published:** 2026-06-16

**Authors:** Lin Yuan, Weixue Yuan, Jiaxin Han, Ge Wang, Jie Jia, Wenqi Xu, Shuang Wang, Shuang Bi, Menglei Xia, Lijuan Ma

**Affiliations:** 1 Key Laboratory of Industrial Fermentation Microbiology Ministry of Education, Tianjin Key Laboratory of Industrial Microbiology, College of Biotechnology, Tianjin University of Science and Technology, Tianjin 300457, China; yuanlin@tust.edu.cn (L.Y.); yuanweixue_1@163.com (W.Y.); 15713577312@163.com (J.H.); 15081421681@163.com (G.W.); 15376188460@163.com (J.J.); xwq270801@163.com (W.X.); m15031563205@163.com (S.W.); mlxia@tust.edu.cn (M.X.); 2 Beijing Engineering and Technology Research Center of Food Additives, Beijing Advanced Innovation Center for Food Nutrition and Human Health, School of Food and Health, Beijing Technology and Business University (BTBU), Beijing 100048, China

**Keywords:** lytic polysaccharide monooxygenases, computer-aided, directed modification, lignocellulose degradation, molecular dynamics simulations

## Abstract

Lytic polysaccharide monooxygenases (LPMOs) are promising enzymes for lignocellulose degradation; however, wild-type LPMOs often exhibit limited catalytic activity and stability. In this study, computer-aided virtual saturation mutagenesis was applied to *An*LPMO15g from *Aspergillus niger*, and eight potentially beneficial mutants (S197H, S197F, E185V, E185L, E185M, E185I, Q108M, and A249P) were identified based on predicted changes in unfolding free energy (∆∆G). Six mutants demonstrated enhanced activity in a 2,6-dimethoxyphenol (2,6-DMP) oxidation assay, which serves as a proxy for peroxidase-like activity. The E185V mutant exhibited a 45% increase over the wild type. The triple mutant E185V/Q108M/A249P further increased the catalytic efficiency by 56%. Notably, when combined with cellulase, E185V/Q108M/A249P enabled a 202.5% increase in reducing sugars from wheat straw, achieving a synergy degree of 1.83, highlighting its potential to improve agricultural residue conversion. Molecular dynamics simulation suggested that the E185V/Q108M/A249P triple mutant induced flexible conformational changes in six residues, which may improve substrate binding affinity. This study presents an effective strategy for engineering AA9 family LPMOs to enhance catalytic performance, facilitating efficient and cost-effective degradation of lignocellulosic biomass with implications for sustainable agricultural waste management and circular bioeconomy.

## 1. Introduction

Lytic polysaccharide monooxygenases (LPMOs) are copper-dependent redox enzymes that cleave the glycosidic bonds in different polysaccharides through oxidation [1]. In 2010, Vaaje-Kolstad et al. [2] first reported that the oxidative cleavage of polysaccharides by a non-catalytic protein, CBP21, originally discovered in *Serratia marcescens*, significantly promoted chitin hydrolysis. This discovery was followed by the identification of similar enzymes, previously classified as CBM33 and GH61, that exhibited comparable catalytic performance [2,3,4]. These enzymes were subsequently redefined as LPMOs and categorized as “auxiliary activities” (AA) enzymes in the CAZy database [5]. LPMOs are widely found in fungi, bacteria, archaea, marine organisms, and even viruses. To date, more than 80 kinds of LPMOs have been discovered, classified into eight families (AA9, 10, 11, 13, 14, 15, 16, and 17) based on their amino acid sequences, which predominantly target chitin, starch, cellulose, hemicellulose, and pectin [2,6,7,8,9,10,11]. Among them, the AA9 family of LPMOs has gained significant attention for their ability to disrupt the crystal structure of cellulose, thereby improving the cellulase accessibility and enhancing cellulose hydrolysis [12,13]. From an agricultural perspective, such properties are critical for unlocking the value of crop residues, an abundant yet underutilized resource, supporting the development of sustainable, climate-smart agricultural systems.

LPMOs typically consist of multiple domains, with the catalytic module located at the N-terminus for most LPMOs. This module forms a β-sandwich structure composed of 8–10 typical β folds, connected by some loops [7,14]. Notably, long and variable loops form the active center, which contains two conserved histidine residues that coordinate a copper ion, along with a tyrosine (for AA9) or phenylalanine (for AA10), forming the “histidine brace” [15,16]. The general catalytic mechanism involves LPMOs binding to polysaccharide substrates, accepting electrons from electron donors, and transferring these electrons to Cu(II) in the active site, reducing it to Cu(I). Cu(I) then binds and activates oxygen, oxidatively cleaving the glycosidic bond, generating both oxidized and non-oxidized products [17,18]. However, the diverse modular composition of LPMOs results in different substrate regioselectivity and cleavage positions, which have been reported at the C1 position of the glucosidic bond (leading to the production of aldonolactone), C4 position (generating 4-ketoaldose), or at the C1/C4 or possibly even the C6 position [19,20]. Additionally, H_2_O_2_ has been identified as the preferred co-substrate, or even the only co-substrate, for LPMOs, capable of driving the reaction in the absence of O_2_. The concentration of H_2_O_2_ significantly affects the activity of LPMOs [21,22].

Numerous studies have demonstrated that AA9 family LPMOs enhance the degradation of cellulose, hemicellulose, and lignocellulose when combined with glycoside hydrolases. A clear synergistic effect has been found between LPMOs and commercial cellulases such as Cellic^®^Ctec2 and Celluclast^®^. For example, the hydrolysis rate of wheatgrass increased by 54% when *Gt*LPMO was used in combination with Celluclast^®^ 1.5 L and *Gt*Xyn10A [23]. Similarly, the combination of *Af*AA9_B from *Aspergillus fumigatus* with Celluclast^®^ 1.5 L enhanced the hydrolysis rate of Avicel^®^ PH-101 and bagasse by 18% and 22%, respectively [24]. In our previous study, an AA9 family LPMO from *A. niger* (*An*LPMO15g) was found to efficiently cleave glycosidic bonds by oxidation of the C1 position, releasing nearly twice as much reducing sugar when acting on unpretreated rice straw powder in synergy with commercial cellulase (Cellic^®^CTec2) [25]. Notably, *An*LPMO15g also increased reducing sugar release by 30% in the absence of an external electron donor, suggesting that lignin or its degradation products may act as the electron donors, as proposed in earlier research [26]. These findings highlight the potential of *An*LPMO15g for lignocellulosic biomass degradation. However, the catalytic efficiency and thermal stability of LPMOs from natural sources usually struggle to meet the requirements of industrial applications, limiting their effectiveness in lignocellulose degradation. Given the global need for sustainable agricultural intensification and the valorization of crop residues, it is therefore imperative to develop robust AA9 LPMOs with improved catalytic activity and stability through microbial biotechnology approaches.

Unlike our previous study on the C293F mutant of the same enzyme, *An*LPMO15g, which used error-prone PCR and Chou-Fasman prediction [27], the present study employs a FoldX-guided virtual saturation mutagenesis strategy that systematically evaluates ΔΔG across multiple sites. This approach led to the identification of a novel triple mutant (E185V/Q108M/A249P) with superior catalytic efficiency and synergy with cellulase, which has not been reported before. The aim of this study is to develop a computational-experimental pipeline to enhance LPMO performance and to elucidate the structural basis for the improved activity using molecular dynamics simulations. This work provides a rational strategy for engineering biomass-degrading enzymes and supports microbial innovation for sustainable agriculture and circular resource management.

## 2. Materials and Methods

### 2.1. Strains and Enzymes

The gene encoding *An*LPMO15g was cloned from *A. niger* CBS 513.88 (CBS, Institute of the Royal Netherlands Academy of Arts and Sciences, Utrecht, The Netherlands). The vector pPICZαA and *Pichia pastoris* GS115 were used for the heterologous expression of *An*LPMO15g. *Escherichia coli* DH5α was used for cloning the recombinant plasmids. Cellulase (Cellic^®^ CTec2) was provided by Novozymes (China) Biotechnology Co., Ltd. (Tianjin, China). All chemicals used in this study were of analytical grade and purchased from Sigma-Aldrich (St Louis, MO, USA).

### 2.2. Bioinformatics Analysis

Sequence alignment was performed using ClustalW within MEGA 7.0. ENDscript/ESPript 2.0 (https://endscript.ibcp.fr/ESPript/ENDscript/) (accessed on 1 January 2023) was used to analyze the sequence alignment result. SWISS-MODEL (https://swissmodel.expasy.org/) (accessed on 1 January 2023) was used to generate the 3D structures of *An*LPMO15g and its mutants. PyMOL 2.5 software was employed for visualizing the protein structures.

### 2.3. Prediction of Mutation Site on AnLPMO15g Using FoldX

The effects of mutagenesis on unfolding free energy (ΔΔG) were simulated and predicted using FoldX, based on the protein’s bioinformation. In this study, *Ls*AA9A (PDB ID: 5NLN) from *Lentinus similis* with a known crystal structure was used as a template to construct the 3D structure of *An*LPMO15g using SWISS-MODEL (https://swissmodel.expasy.org/) (accessed on 1 January 2023). Potential mutation sites on *An*LPMO15g were virtually saturated with mutations by FoldX 5.0 (https://foldxsuite.crg.eu/) (accessed on 1 January 2023). The change in ΔΔG for each mutant protein relative to the wild type (WT) *An*LPMO15g was calculated to identify candidate mutations that could enhance the thermal stability of the protein. ΔΔG was determined as the difference between the unfolding free energy of the mutant (ΔG_mutant_) and that of the wild-type (ΔG_WT_). A threshold of ΔΔG < −10 kJ/mol (−2.39 kcal/mol) was selected based on FoldX recommendations for mutations likely to confer measurable thermal stabilization. To improve screening efficiency, computational results were filtered using this criterion. Furthermore, mutation sites within the catalytic active center and conserved regions of *An*LPMO15g were excluded, based on sequence alignment (Figure S1) and molecular docking results (Figure S2), to avoid interference with the protein’s functional properties.

The 3D structure of *An*LPMO15g was modeled using SWISS-MODEL with *Ls*AA9A (PDB ID: 5NLN) as the template. The template shared 52.35% sequence identity with *An*LPMO15g. The model quality was assessed by QMEAN (−0.73) and GMQE (0.50), indicating acceptable reliability. All structural figures were prepared using PyMOL 2.5.

### 2.4. Construction of Recombinant Strains

The recombinant strain of *An*LPMO15g was constructed through site-directed mutagenesis. The primers used for single-point mutation were listed in Table 1. The *An*LPMO15g plasmid served as the template for amplifying the mutated target genes through reverse PCR. Reverse PCR conditions were as follows: initial denaturation at 95 °C for 5 min; 30 cycles of denaturation at 95 °C for 30 s, annealing at 68 °C for 7 min, and extension at 72 °C for 10 min, followed by a final extension at 72 °C for 10 min. The PCR products were treated with DpnI for 1 h at 37 °C to digest the parental plasmids. The treated PCR products were then transformed into *E. coli* and positive clones were verified by colony PCR and DNA sequencing (Genewiz, Beijing, China).

### 2.5. Production, Purification, and Assay of the Recombinant AnLPMO15g

The sequenced recombinant plasmids were extracted using a plasmid extraction kit (Vazyme, Beijing, China) and electrotransformed into *P. pastoris*. The *P. pastoris* recombinants were cultured for enzyme production as described in our previous study [25]. A 0.5 mL aliquot of the culture was added to 50 mL of buffered glycerol-complex medium with yeast extract (BMGY) and incubated in a shaker at 30 °C and 220× *g* for 12–16 h to achieve logarithmic growth phase (OD_600 nm_ = 2). The yeast cells were harvested by centrifugation (7000× *g*, 5 min), resuspended in buffered methanol-complex medium with yeast extract (BMMY), and cultured for 120 h at 30 °C and 240× *g*. Methanol was added daily for five consecutive days to a final concentration of 0.5% (*v*/*v*), and the culture was shaken at 220× *g* and 30 °C. After 5 days of induction, the fermentation broth was transferred to a 50 mL centrifuge tube, centrifuged at 4 °C and 8000× *g* for 10 min, and the supernatant was collected. The culture supernatant was filtered through a 0.22 μm filter and loaded onto the Ni-Agarose Resin for 6 × His-tagged proteins (CWBIO, Beijing, China). The bound proteins were eluted with 80 mM imidazole, 20 mM Tris–HCl (pH 7.9), and 0.5 mM NaCl. Finally, the collected proteins were concentrated using ultrafiltration tubes (30 kDa molecular-weight cut-off, GE Healthcare, Shanghai, China) by centrifugation at 4000× *g* for 20 min in a pH 5.0, 50 mM sodium acetate buffer. The assay of *An*LPMO15g and its mutants, including SDS-PAGE analysis and protein concentration determination, was performed as described in our previous study [25].

### 2.6. Determination of Enzyme Activity

The activity of *An*LPMO15g and its mutants was measured using 2,6-DMP as the chromogenic substrate and hydrogen peroxide (H_2_O_2_) as the co-substrate [28]. The 1 mL reaction mixture comprised 860 μL of 116 mM, pH 7.5 phosphate buffer, 100 μL of 10 mM 2,6-DMP solution, 20 μL of 5 mM H_2_O_2_ stock solution, and 10 μg of purified *An*LPMO15g or its mutant. The reaction was carried out at the specified temperature for 5 min, and the absorbance at 469 nm was determined. Enzyme activity (U) was defined as the amount of enzyme required to produce 1 μmol of oxidation products per minute under the given reaction conditions. The enzyme activity was calculated using the following formula:

(1) $$X=\frac{N\times A\times V_{1}\times 10^{9}}{\varepsilon_{469}\times b\times t\times C\times V_{2}}$$

where X (U/g) is the specific enzyme activity, A is the change in absorbance, V_1_ (mL) is the reaction volume, C (mg/mL) is the protein concentration, V_2_ (μL) is the protein volume, N is the dilution factor, ε_469_ (53,200 L·mol^−1^·cm^−1^) is the molar absorption coefficient of quinone, b (cm) is the optical path length, and t (min) is the reaction time.

The optimal temperature, thermal stability, and kinetic constants for *An*LPMO15g and its mutants were determined using the 2,6-DMP assay method [28]. The relative enzyme activity of *An*LPMO15g and its mutants was measured within the optimal temperature range of 30–80 °C according to the protocol described above.

The stability of *An*LPMO15g and its mutants was assessed by incubating the enzymes in a water bath at 40 °C. Samples were taken at different time points, and the residual activities of *An*LPMO15g and its mutants were subsequently measured within 12 h under optimal conditions, employing the standard protocol described previously. The kinetic parameters, *K_m_* and *V_max_*, for *An*LPMO15g and its mutants were determined by conducting reactions with eight different concentrations of 2,6-DMP (1–40 mM) at 65 °C for 5 min in a 50 mM sodium phosphate buffer (pH 7.5). A control reaction without an enzyme was also performed under the same conditions.

Although the 2,6-DMP/H_2_O_2_ assay is convenient for rapid screening, it measures a peroxidase-like oxidation of a soluble chromogenic substrate rather than direct oxidative cleavage of polysaccharides [28]. Therefore, this assay was used only as a comparative screening method for the mutants. All conclusions regarding lignocellulose degradation were cross-validated using reducing sugar assays on cellulosic and lignocellulosic substrates (Section 2.7).

### 2.7. Hydrolysis Activity on Different Cellulosic Substrates

The enzyme activity of *An*LPMO15g and its mutants was measured under pH 5.0 and 50 °C using 1% (*w*/*v*) microcrystalline cellulose, cellohexaose (Cell6), carboxymethyl cellulose (CMC), and xylan as substrates. The 1 mL reaction mixture included 0.01 g of substrate, 1 mg of purified protein (100 mg protein/g substrate), 1 mM of ascorbic acid, and 50 mM sodium acetate buffer (pH 5.0). For cellulase synergy experiments, Cellic^®^ CTec2 was loaded at 1.1 FPU/g substrate. The reaction mixtures were incubated for 48 h at 50 °C with shaking at 200× *g* on an orbital shaker. The reactions were subsequently terminated by boiling for 5 min. The concentrations of reducing sugars in each reaction mixture were determined using the 3,5-dinitrosalicylic acid (DNS) method [29].

To assess the synergistic hydrolysis effect of *An*LPMO15g and its mutant with cellulase, a cellulase reaction system was used. The 1 mL of reaction system contained 1 mg of purified recombinant protein (*An*LPMO15g or its mutants), 1.1 FPU/mL of Cellic^®^ CTec2, 0.01 g of substrate prepared with 50 mM sodium acetate buffer (pH 5.0), and 1 mM ascorbic acid as a reducing cofactor in a 1.5 mL EP tube. The reaction was carried out at 50 °C with shaking at 200× *g* for 48 h. After the reaction, the mixture was boiled for 5 min to terminate the reaction. The sediment was then removed by centrifugation at 13,000× *g* for 1 min, and the supernatant was collected. The concentration of reducing sugars was then determined using the DNS method [30]. The synergistic ability of *An*LPMO15g (or its mutant) with cellulase was assessed by calculating the synergy degree, using the following formula:

(2) $$\mathrm{Synergy}\;\mathrm{degree}=\frac{C_{A+C}}{C_{c}+C_{A}}$$

where C_A_ (g/L) is the reducing sugar yield obtained by *An*LPMO15g (or its mutant) acting on the substrate alone, C_C_ (g/L) is the reducing sugar yield obtained by cellulase alone, and C_A+C_ (g/L) is the reducing sugar yield obtained by *An*LPMO15g (or its mutant) and cellulase together. A synergy degree greater than 1 indicates a synergistic relationship between the two enzymes, a value of 1 represents an additive effect, and a value less than 1 indicates an antagonistic relationship.

### 2.8. MD Simulation

In this study, Amber 22 was employed for MD simulation. The composite structures of *An*LPMO15g and its mutants in complex with Cell6 were obtained through molecular docking. The ff19SB force field was used to model bonded and non-bonded interactions of proteins. The system was solvated in a cubic box of explicit TIP3P (Transferable Intermolecular Potential with 3 Points) water molecules with a 10 Å buffer region, and counterions were added to neutralize the system. Following energy minimization, the system was gradually heated from 0 K to 310 K over a period of 500 picoseconds. The system was then equilibrated in the canonical ensemble (NVT) at 310 K for preliminary equilibration. Subsequently, a 100-ns MD simulation was conducted in an isothermal-isobaric ensemble (NPT) while maintaining periodic boundary conditions. All covalent bonds involving hydrogen atoms were constrained using the SHAKE algorithm throughout the simulation. The MD simulations focus solely on substrate binding and do not include oxygen or hydrogen peroxide.

### 2.9. Statistical Analysis

Data are presented as mean ± standard deviation (SD) from three replicates. Prior to parametric analysis, normality and homogeneity of variance were assessed using the Shapiro–Wilk and Levene’s tests, respectively. Differences between the two groups were analyzed using Student’s *t*-test. Comparisons among three or more groups were evaluated using one-way analysis of variance (ANOVA), followed by Tukey’s honestly significant difference (HSD) post hoc test when significant differences were detected (*p* < 0.05). When the assumptions for ANOVA were not met, the non-parametric Kruskal–Wallis test was used. For kinetic analysis, initial reaction rates at each substrate concentration were measured in triplicate. Kinetic parameters (*K_m_* and *V_max_*) were obtained by non-linear regression fitting of the Michaelis–Menten equation using GraphPad Prism version 9.0 (San Diego, CA, USA) software. The reported values represent the fitted parameters ± SD derived from triplicate measurements. All statistical analyses were performed using SPSS version 22.0 (IBM SPSS Inc., Chicago, IL, USA). Differences were considered statistically significant at *p* < 0.05.

## 3. Results and Discussion

### 3.1. Identification of Potential Sites for Directed Mutagenesis

Based on the negative correlation between the ΔΔG and thermal stability, mutation sites that significantly decreased the ΔG of the WT protein molecules were selected as candidate sites for molecular modification. A total of 14 mutations meeting the criterion of ΔΔG < −2.39 kcal/mol were identified using the FoldX tool, as shown in Table 2. In order to enhance the thermal stability of *An*LPMO15g without affecting its catalytic properties and functions, mutations corresponding to amino acid sites near the catalytic active center and in conserved regions were excluded. According to the 3D structure of *An*LPMO15g (Figure S3) and multiple sequence alignment results (Figure S1), mutations at S46, N64, A180, V187, and I229 were excluded, as these amino acid sites are located in conserved regions of *An*LPMO15g (Figure S1) and generally have important physiological or genetic functions. Additionally, residues within 5 Å of the copper active site (based on molecular docking results in Figure S2) were also excluded to avoid direct interference with catalytic activity. Consequently, eight mutants identified as S197H, S197F, E185V, E185L, E185M, E185I, Q108M, and A249P were selected for further experimental verification. The introduced amino acids are predominantly hydrophobic: E185V (negatively charged glutamate replaced by hydrophobic valine), Q108M (polar glutamine replaced by hydrophobic methionine), and A249P (alanine replaced by proline, which may increase backbone rigidity), which may enhance protein stability and substrate binding.

### 3.2. Mutant Protein Expression and Purification

The SDS-PAGE results of mutant protein validations are shown in Figure S4, indicating that the bands for all purified AnLPMO15g mutants were approximately 66 kDa, which is consistent with the theoretical molecular weight of the mutants (S197H, S197F, E185V, E185L, E185M, E185I, Q108M, and A249P). The protein yield for the triple mutant E185V/Q108M/A249P was 1.52 ± 0.07 mg/L (pPIC9K), 1.48 ± 0.06 mg/L (pPICZαA), and 1.65 ± 0.08 mg/L (double plasmid). Yields for other mutants were not individually quantified, but SDS-PAGE showed comparable expression levels (Figure S4). This indicates successful expression of all mutants, which were subsequently used for further enzymatic property studies.

### 3.3. Optimal Temperature and Thermal Stability of Mutants

**Figure 1:**
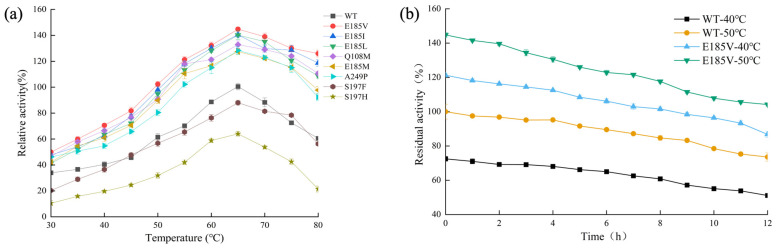
Optimal reaction temperature for *An*LPMO15g and the mutants (**a**). Thermal stability of *An*LPMO15g and mutant E185V at 40 °C and 50 °C (**b**).

In order to compare the optimal temperature of the mutants, enzyme activity was measured using 2,6-DMP as the substrate, with the WT protein serving as the control. The experimental temperature range was set from 30 to 80 °C. As shown in Figure 1a, all mutants exhibited maximum activity at 65 °C, which matched the optimal temperature of the WT enzyme. Notably, the enzyme activities of E185V, E185I, E185L, Q108M, E185M, and A249P were higher than those of the WT, suggesting an increase in catalytic activity due to the mutations. This enhancement may be attributed to the fact that the mutated amino acids are predominantly hydrophobic, which could increase the protein’s hydrophobicity and consequently enhance its catalytic activity. Among these mutants, E185V exhibited approximately 45% higher enzyme activity than the WT and demonstrated the highest activity among all mutants, thus emerging as the most promising mutant. This proxy activity prompted us to further evaluate its performance on cellulosic and lignocellulosic substrates (Section 3.5 and Section 3.6).

To assess the thermal stability of E185V, the residual enzyme activity was measured after incubation at 40 °C and 50 °C for 0–12 h (Figure 1b). At 40 °C, after 12 h, the WT retained 67% of its residual enzyme activity, while the mutant retained over 75%, indicating an improvement in thermal stability. At 50 °C, after 12 h, the WT retained approximately 75% of its initial activity, whereas E185V retained approximately 78%. These results demonstrate that E185V exhibits significantly better thermal stability than the WT at both 40 °C and 50 °C, the latter being the temperature used for cellulosic substrate hydrolysis. The future work will include stability assessments under optimal conditions. In addition, the 2,6-DMP/H_2_O_2_ assay used in this section measures a peroxidase-like activity and does not directly reflect oxidative cleavage of polysaccharides [31,32]. Therefore, while this assay was useful for rapid screening and kinetic parameter determination (Table 3), the enhanced lignocellulose degradation claimed in this study is solely based on reducing sugar assays on cellulosic and lignocellulosic substrates (Section 3.5 and Section 3.6).

### 3.4. Combinatorial Mutagenesis for Further Improvement in Catalytic Efficiency

**Figure 2:**
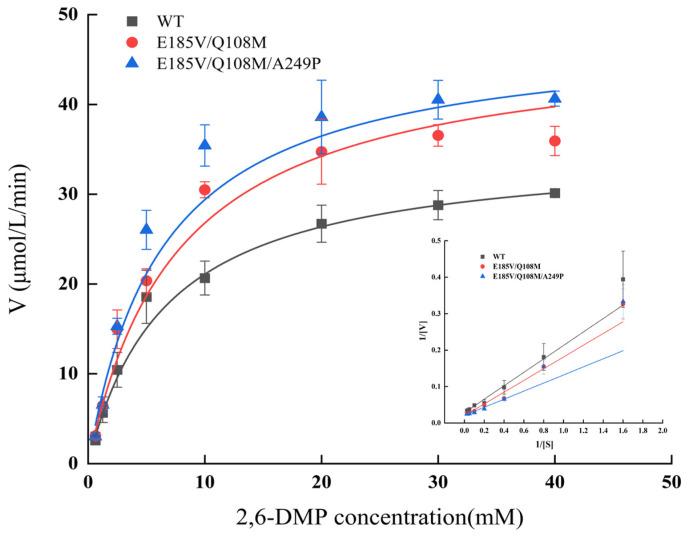
Enzyme activities of *An*LPMO15g and mutants at different concentrations of 2,6-dimethoxyphenol. Michaelis–Menten kinetic parameters of *An*LPMO15g and the mutants were calculated based on the results.

In order to further enhance the catalytic efficiency and thermal stability, combinatorial mutagenesis was performed on the mutants that showed improved catalytic performance. Two-point and three-point combined mutants, E185V/Q108M, E185V/A249P, Q108M/A249P, and E185V/Q108M/A249P were designed based on the previously identified mutants E185V, Q108M, and A249P. The catalytic efficiency of these combinatorial mutants was measured using different concentrations of 2,6-DMP as the substrate. The maximum reaction rate (*V_max_*) and Michaelis constant (*K_m_*) for both the WT and the mutants were determined by fitting the Michaelis–Menten equation. As shown in Figure 2 and Table 3, the *V_max_* values were as follows: 42.52 U/g for E185V/Q108M, 47.64 U/g for E185V/Q108M/A249P, and 33.98 U/g for the WT. The *K_m_* values were 5.05 mM for E185V/Q108M, and 4.84 mM for E185V/Q108M/A249P, whereas WT had a *K_m_* value of 5.41 mM. For comparison, previous reports showed *K_m_* values of 245 mM for *Nc*LPMO9C and 23.7 mM for *Mt*LPMO9G [28,30], indicating that *An*LPMO15g has a stronger substrate affinity.

The catalytic efficiency (*kcat/K_m_*) of E185V/Q108M was 4.10 s^−1^·mM^−1^, which was approximately 49% higher than that of the WT (2.76 s^−1^·mM^−1^). The triple mutant E185V/Q108M/A249P showed a further increase to 4.31 s^−1^·mM^−1^, representing a 56% improvement over the WT and 22% higher than that of the C293F mutant, which exhibited the best performance in the initial stage of single-point mutagenesis [27]. These results indicate that both combinatorial mutants possess enhanced catalytic efficiency, with the triple mutant being the most effective. Their actual lignocellulose-degrading performances were then assessed by reducing sugar release from various substrates (Section 3.6).

### 3.5. Catalytic Activity on Different Substrates

**Figure 3:**
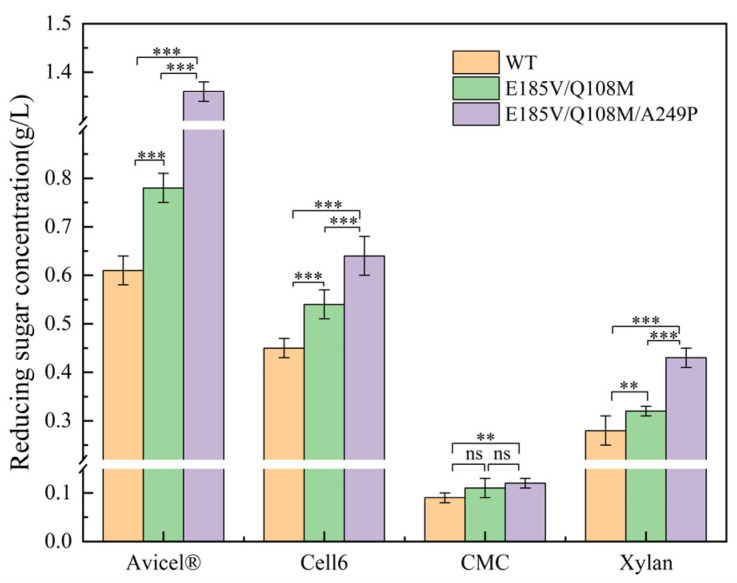
Reducing sugar concentration of *An*LPMO15g and mutants when acting on different substrates (Avicel^®^, CMC, Cell6, and xylan). Data are presented as mean ± SD (*n* = 3). Statistical significance was determined by one-way ANOVA followed by Tukey’s HSD post hoc test (*** *p* < 0.001, ** *p* < 0.01), and “ns” indicates no significant difference (*p* > 0.05).

In order to evaluate the catalytic activity of *An*LPMO15g and its mutants on various substrates with different structural characteristics, hydrolysis was conducted using Avicel^®^, CMC, Cell6, and xylan. As shown in Figure 3, both E185V/Q108M and E185V/Q108M/A249P exhibited a significant increase (*p* < 0.01) in reducing sugar yield compared to *An*LPMO15g across all substrates. The reducing sugar yields of *An*LPMO15g and the mutants were higher when they acted on Cell6 and Avicel^®^ than on CMC and xylan. When acting on Avicel^®^, mutants E185V/Q108M and E185V/Q108M/A249P exhibited reducing sugar yield of 0.78 g/L and 1.36 g/L, respectively, representing highly significant increases (*p* < 0.001) of 27.8% and 122.9% compared to *An*LPMO15g (0.61 g/L). The reducing sugar yields from Cell6 catalyzed by E185V/Q108M and E185V/Q108M/A249P were 0.54 g/L and 0.64 g/L, showing highly significant increases (*p* < 0.001) of 20.0% and 42.2%, respectively, compared to *An*LPMO15g. These results suggest that despite the recalcitrant crystal structure of Avicel^®^, it remains the most suitable substrate for *An*LPMO15g and its mutants among the tested four substrates. This supports previous studies showing the ability of AA9 LPMOs to disrupt the crystal structure of cellulose effectively [33,34,35,36].

### 3.6. Synergistic Effect with Cellulase on Different Lignocellulosic Substrates

**Figure 4:**
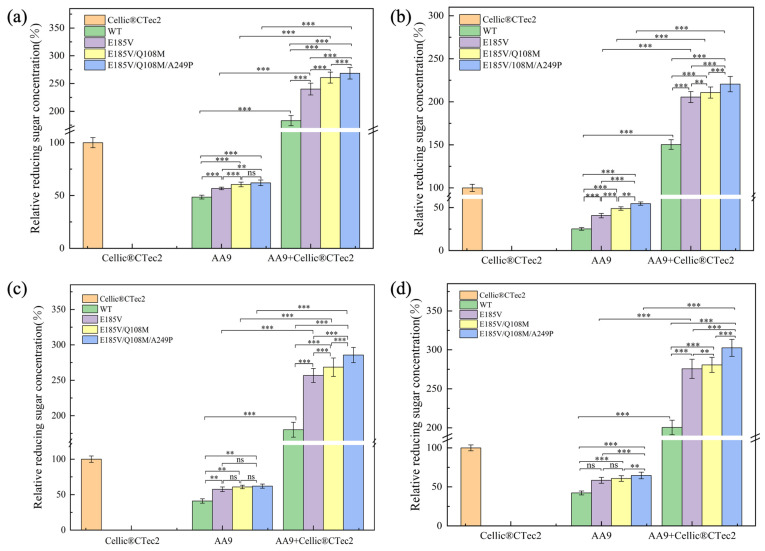
Relative reducing sugar concentration produced by *An*LPMO15g and mutants in concert with cellulase on different substrates. (**a**) Avicel^®^, (**b**) Corn cob, (**c**) Grass powder, (**d**) Wheat straw. Data are presented as mean ± SD (*n* = 3). Statistical significance was determined by one-way ANOVA followed by Tukey’s HSD post hoc test (*** *p* < 0.001, ** *p* < 0.01), and “ns” indicates no significant difference (*p* > 0.05).

The synergistic effect of *An*LPMO15g and its mutants with cellulase was evaluated in degradation experiments using Avicel^®^, corn cob, grass powder, and wheat straw as substrates. The concentration of reducing sugar obtained by cellulase alone was considered as 100%, and the results for *An*LPMO15g or its mutants alone and the combination with cellulase were expressed as relative values. As shown in Figure 4, *An*LPMO15g and its mutants significantly increased the yield of reducing sugar when acting together with cellulase across different substrates, exhibiting clear synergistic effects. Among them, mutant E185V/Q108M/A249P showed the best performance. When acting on Avicel^®^ and corn cob, the reducing sugar yield from E185V/Q108M/A249P combined with cellulase was 168.5% and 120.5% higher, respectively, compared to cellulase alone, with synergy degrees of 1.65 and 1.43 (Figure 4a and Figure 4b). On grass powder and wheat straw, the yield increased by 185.6% and 202.5%, with synergy degrees of 1.76 and 1.83, respectively (Figure 4c and Figure 4d). The results showed that mutant E185V/Q108M/A249P exhibited better performance in the synergistic degradation of lignocellulosic biomass compared to the mutant C293F reported in our previous study [27]. Notably, the synergistic effect of E185V/Q108M/A249P and cellulase was more pronounced with grass powder and wheat straw, possibly due to the higher lignin content of these two substrates compared to Avicel^®^ and corn cob. Lignin has been reported to serve as an electron donor for LPMOs [37], which may enhance the synergistic effect. In addition, the structure of lignocellulosic substrate and the content of hemicellulose component may also affect the synergistic effect of LPMOs in the hydrolysis of lignocellulose. However, whether the observed improvements are maintained at larger reaction volumes remains to be tested in future scale-up studies.

### 3.7. MD Simulation of Substrate Binding

#### 3.7.1. Root Mean Square Deviation and Root Mean Square Fluctuation Analysis

**Figure 5:**
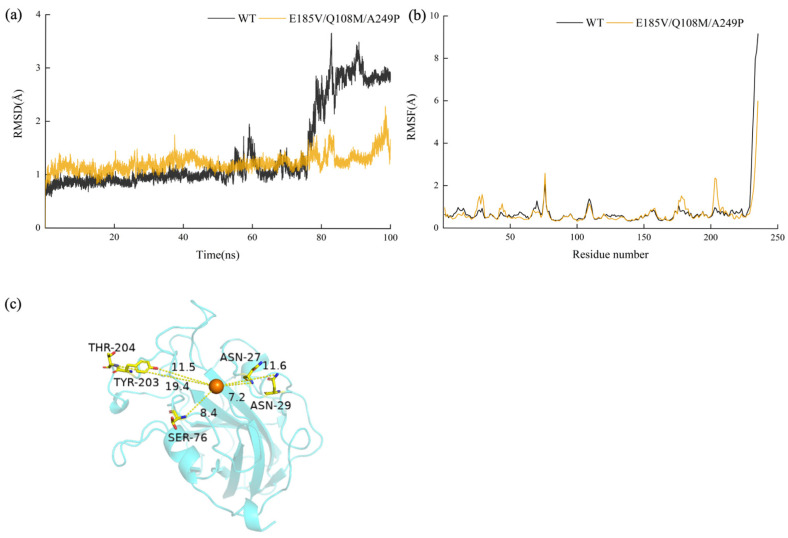
Molecular dynamics simulation of the enzyme-Cell6 complex during the simulation. (**a**) Root mean square deviation (RMSD) of WT-Cell6 (black) and E185V/Q108M/A249P-Cell6 (yellow). (**b**) Root mean square fluctuation (RMSF) of WT-Cell6 (black) and E185V/Q108M/A249P-Cell6 (yellow). (**c**) The amino acid residues with significant fluctuation in E185V/Q108M/A249P and their distance (Å) to the copper ion.

The root mean square deviation (RMSD) is a key metric for assessing the positional shift of the overall conformation of the enzyme-Cell6 complex during the simulation. This analysis was focused on the catalytic structural domain of the enzyme. As shown in Figure 5a, the RMSD value of the WT-Cell6 complex fluctuated around 1 Å during the first 80 ns of the simulation, then rapidly increased to 3 Å, and finally steadily fluctuated around 3 Å. In contrast, the RMSD of the E185V/Q108M/A249P-Cell6 complex showed less variation, consistently fluctuating within the range of 1 Å to 2 Å. Both groups reached a relatively stable state when the simulation was run for 100 ns. Root mean square fluctuation (RMSF) is able to accurately express the change of each atom relative to its average position. It characterizes the average change of the structure over time, and can provide information such as the stability of protein flexible regions, especially key amino acid residues. As shown in Figure 5b, significant RMSF fluctuations were observed near residues N27, N29, S76, T179, Y203, and T204 in the E185V/Q108M/A249P-Cell6 model (corresponding to S49, S51, S101, S197, I220, and Y221 of E185V/Q108M/A249P). These residues exhibit flexibility during the simulation, suggesting that they may play a crucial role in improving the enzyme activity. Based on its 3D structure, further analysis of the results can be seen in Figure 5c. Except for threonine at position 179, these residues are mostly located in the loop regions of the mutant’s active surface. The introduction of mutation sites increased the flexibility of the loop ring in the enzyme’s active center, making it more flexible to bind to the substrate, thereby improving its catalytic efficiency. This supports the hypothesis that the loop region of the LPMO active surface is involved in substrate binding.

#### 3.7.2. Radius of Gyration and Hydrogen Bonding Analysis

**Figure 6:**
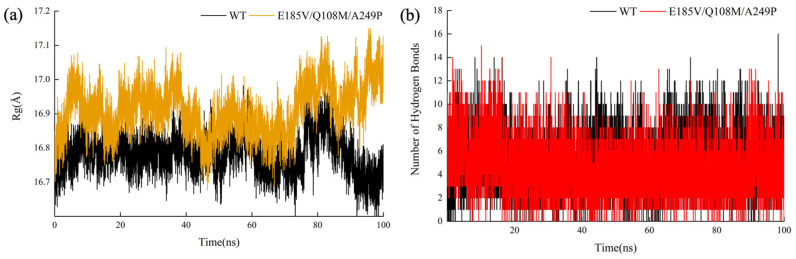
Comparison of changes in the radius of gyration (Rg) (**a**) and the number of hydrogen bonds (**b**) between WT-Cell6 complex and E185V/Q108M/A249P-Cell6 complex during a 100 ns simulation.

The radius of gyration (Rg) was used to assess changes in the spatial conformation of the WT-Cell6 and E185V/Q108M/A249P-Cell6 complexes during the 100 ns simulation. A larger Rg value indicates a looser protein conformation, while a smaller value suggests a denser and more stable conformation. As shown in Figure 6a, the Rg value of the WT-Cell6 complex remained stable around 16.8 Å throughout the entire simulation process. In contrast, the Rg value of E185V/Q108M/A249P-Cell6 complex was consistently higher, especially between 0–40 ns at the beginning of the simulation and 80–100 ns at the end of the simulation, where the value reached approximately 17.1 Å. This indicates that the spatial conformation of the mutant changes from a tight to a loose state during these two time periods, with increased flexibility. Overall, mutant E185V/Q108M/A249P had a loose conformation, more flexible loops, and easier substrate binding, which led to an increase in catalytic activity and efficiency. Additionally, the hydrogen bonding analysis (Figure 6b) revealed that the E185V/Q108M/A249P-Cell6 complex formed a higher number of hydrogen bonds than the WT-Cell6 complex, indicating a more stable overall structure. These findings suggest that the structural changes observed in Rg and hydrogen bonds are consistent with the results from the RMSF analysis, supporting the conclusion that the mutant has improved flexibility and substrate binding ability, which contribute to its higher catalytic efficiency.

Furthermore, the solvent accessible surface area (SASA) analysis (Figure S5) showed that both WT and mutant fluctuated within 500–700 Å^2^ during the simulation, with no significant difference, indicating that the mutations did not substantially alter the overall solvent exposure of the protein. The current MD simulations provide descriptive insights into conformational changes and substrate binding, but do not directly elucidate the catalytic mechanism involving H_2_O_2_ activation or electron transfer at the copper active site. Such processes would require quantum chemical methods or hybrid QM/MM approaches beyond the scope of this study [38]. We also note that the simulations did not include the binding of molecular oxygen or hydrogen peroxide, nor do they account for electron transfer. Therefore, the observed increases in loop flexibility and substrate binding are only hypothesized to contribute to the enhanced catalytic activity; other factors (e.g., redox potential, H_2_O_2_ accessibility) may also play important roles. Quantitative calculations such as MM-GBSA binding free energy or substrate-contact occupancy were not performed, which is a limitation of the current analysis. Crystallographic validation of the AnLPMO15g structure is currently unavailable; the modeled structure is based on 52.35% sequence identity with LsAA9A (PDB 5NLN). Future studies using X-ray crystallography would provide experimental validation of the structural changes observed in MD simulations.

## 4. Conclusions

This study employed computational predictive structure-based methods to enhance the catalytic performance of *An*LPMO15g. The saturation mutation of *An*LPMO15g was simulated using computer-aided design software FoldX, and eight favorable single-point mutants were determined based on the change of ∆∆G. The experimental results demonstrated that six of these mutants showed an improvement in catalytic performance, with the E185V mutant exhibiting a 45% increase in enzyme activity. The combinatorial mutant E185V/Q108M/A249P exhibited a further enhancement in catalytic efficiency, with a 56% increase compared to the WT. In synergistic hydrolysis experiments, E185V/Q108M/A249P, when used in combination with cellulase, showed a marked 202.5% increase in reducing sugar yield from wheat straw, indicating an excellent synergistic effect with a synergistic degree of 1.83. MD simulation revealed that six amino acid residues in the E185V/Q108M/A249P mutant underwent flexible changes, which likely led to a loose conformation, more flexible loops, and increased substrate binding ability. These structural modifications contribute to the mutant’s enhanced catalytic efficiency. Overall, this study provides a valuable strategy for improving the catalytic performance of LPMOs and other enzymes, and it could aid in the development of more efficient enzymatic cocktails for biomass degradation.

## Figures and Tables

**Table 1:** List of primers used for site-directed mutagenesis.

Primer Name	Primer Sequence ^1^
S197H-F	5′-ACGGCTCCCATGAGCTTCCCTCCGGTGTCTCC-3′
S197H-R	5′-AAGCTCATGGGAGCCGTCGGAGGTGACCTTGA-3′
S197F-F	5′-ACGGCTCCTTCGAGCTTCCCTCCGGTGTCTCC-3′
S197F-R	5′-AAGCTCGAAGGAGCCGTCGGAGGTGACCTTGA-3′
E185V-F	5′-CTACATGGTATGTGTCCAGTTCAAGGTCACCTC-3′
E185V-R	5′-GGACACATACCATGTAGAACTGGGCACCACCC-3′
E185L-F	5′-CTACATGTTATGTGTCCAGTTCAAGGTCACCTC-3′
E185L-R	5′-GGACACATAACATGTAGAACTGGGCACCACCC-3′
E185M-F	5′-CTACATGATGTGTGTCCAGTTCAAGGTCACCTC-3′
E185M-R	5′-GGACACACATCATGTAGAACTGGGCACCACCC-3′
E185I-F	5′-CTACATGATATGTGTCCAGTTCAAGGTCACCTC-3′
E185I-R	5′-GGACACATATCATGTAGAACTGGGCACCACCC-3′
Q108M-F	5′-TCCCGTCATGGTCTACATGGCCCCGACGGCCA-3′
Q108M-R	5′-TGTAGACCATGACGGGACCCTTGTGGGAGGAG-3′
A249P-F	5′-ATCTTCCTCCCCAGCTGCTGCTGCTACCACCTC-3′
A249P-R	5′-CAGCTGGGGAGGAAGATCCGGAGCTGGAGCCG-3′

^1^ The mutagenic site is marked in underline.

**Table 2:** List of mutants with the change of unfolding free energy (∆∆G) less than −2.39 kcal/mol screened by FoldX.

Mutants	∆∆G (kcal/mol) ^1^
S197H	−2.53983
S197F	−2.50902
E185V	−2.39397
E185L	−2.71438
E185M	−3.11987
E185I	−3.15412
V187I	−2.72080
S46L	−2.41606
S46M	−2.91359
N64G	−2.79300
Q108M	−3.01390
A249P	−2.56169
A229M	−2.75333
A180P	−2.47343

^1^ ΔΔG was determined as the difference between the unfolding free energy of the mutant (ΔG_mutant_) and that of the wild type (ΔG_WT_), calculated by FoldX 5.0 (https://foldxsuite.crg.eu/) (accessed on 1 January 2023).

**Table 3:** Michaelis–Menten kinetic parameters of *An*LPMO15g and mutants on 2,6-DMP.

Enzymes	*K*_m_ (mM)	*V*_max_ (U·g^−1^)	*kcat* (s^−1^) ^1^	*kcat/K*_m_ (s^−1^·mM^−1^)	References
*An*LPMO15g (WT)	5.41 ± 0.18 ^a^	33.98 ± 1.20 ^d^	14.92 ± 0.52 ^c^	2.76 ± 0.11 ^d^	This study
E185V/Q108M	5.05 ± 0.15 ^b^	42.52 ± 1.35 ^b^	20.70 ± 0.66 ^a^	4.10 ± 0.18 ^b^	This study
E185V/Q108M/A249P	4.84 ± 0.14 ^c^	47.64 ± 1.28 ^a^	20.84 ± 0.60 ^a^	4.31 ± 0.20 ^a^	This study
C293F	4.98 ± 0.01 ^b^	39.94 ± 0.01 ^c^	17.54 ± 0.04 ^b^	3.52 ± 0.02 ^c^	[27]
*Nc*LPMO9C	245 ± 74	270 ± 20	ND	ND	[28]
*Mt*LPMO9G	23.7 ± 3.5	662.2 ± 44.3	ND	ND	[30]

^1^ *kcat* is the catalytic constant, calculated by dividing *V*_max_ by the enzyme concentration. ND indicates no data detected in the literature. Different letters in the same column indicate significant differences at the *p* < 0.05 level as determined by one-way ANOVA followed by Tukey’s HSD post hoc test.

## Data Availability

The original contributions presented in this study are included in the article/Supplementary Materials. Further inquiries can be directed to the corresponding authors.

## Supplementary Materials

The following supporting information can be downloaded at: https://www.mdpi.com/article/10.3390/foods15122178/s1. Figure S1. Sequence alignment of *An*LPMO15g and AA9 family LPMOs. (The blue rectangle shows the catalytically active sites near Cu^2+^ of *An*LPMO15g; yellow dots are mutation sites for *An*LPMO15g; L1, L2, L3, and L4 were Loop rings near the active center). Figure S2. Optimal conformation diagram of AnLPMOI5g docking with cellohexose. (a) the overall picture; (b) 2D schematic diagram. Figure S3. The three-dimensional structure of *An*LPMO15g. Figure S4. SDS-PAGE results of recombinant *Pichia pastoris*. M: Protein Marker, lanes 1, 2, 3, 4, 5, 6, 7, 8 represent recombinant proteins S197H, S197F, E185V, E185L, E185M, E185I, Q108M, and A249P, respectively. No editing was applied beyond standard brightness/contrast adjustment for the whole image. Figure S5. Solvent accessible surface area (SASA) analysis of E185V/Q108M/A249P.

## Abbreviations

The following abbreviations are used in this manuscript:

LPMO	Lytic polysaccharide monooxygenases
AA	Auxiliary activities
2,6-DMP	2,6-Dimethoxyphenol
MD	Molecular dynamics
BMGY	Buffered glycerol-complex medium with yeast extract
BMMY	Buffered methanol-complex medium with yeast extract
CMC	Carboxymethyl cellulose
DNS	3,5-Dinitrosalicylic acid
RMSD	Root mean square deviation
RMSF	Root mean square fluctuation
WT	Wild type
