# Supplementary material for: Computer-Aided Virtual Saturation Mutagenesis Improves the Lignocellulose-Degrading Performance of an Aspergillus niger LPMO
Source: Foods. 2026 Jun 16;15(12):2178. doi: 10.3390/foods15122178 (PMC13297978; doi:10.3390/foods15122178)
Supplement: Supplementary file 1 [file foods-15-02178-s001.zip › foods-4318641-supplementary.pdf]

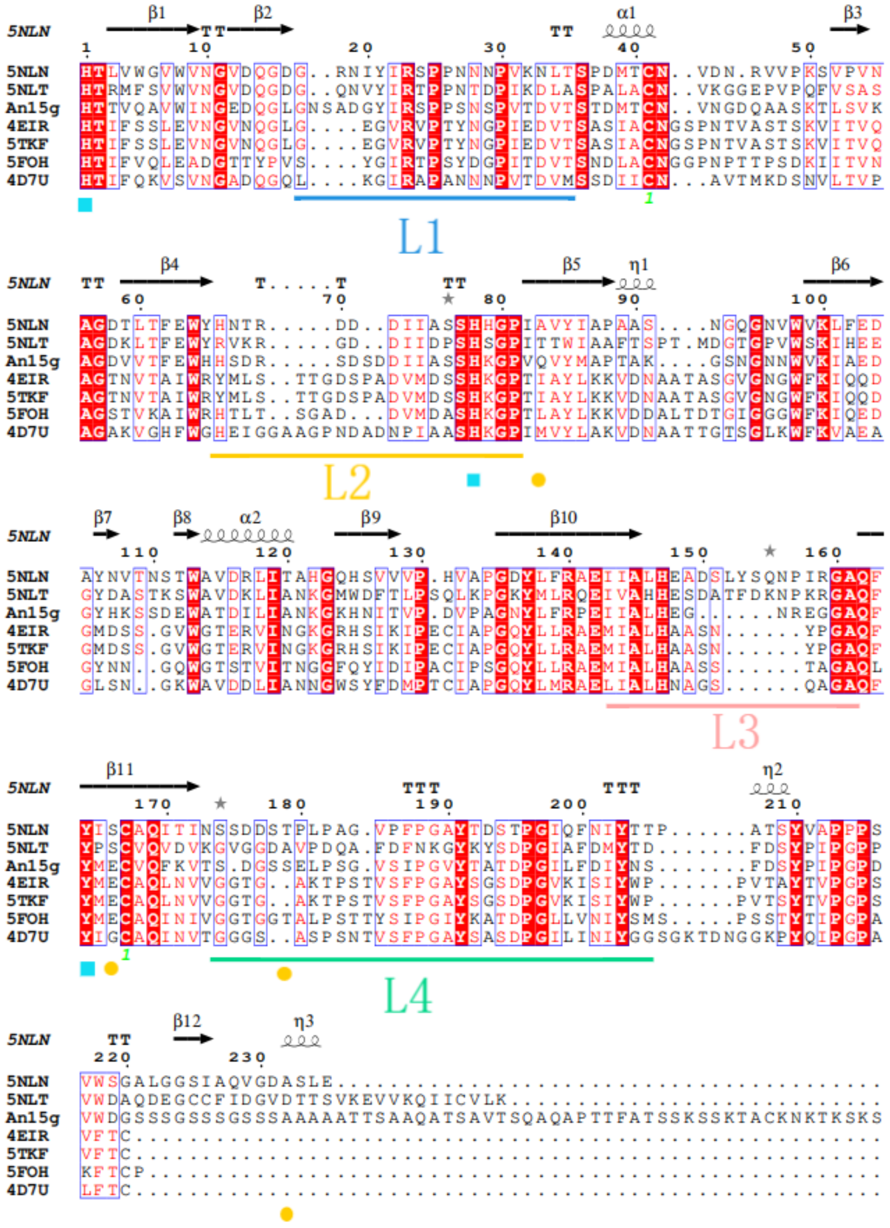

Figure S1. Sequence alignment of *An*LPMO15g and AA9 family LPMOs (The blue rectangle shows the catalytically active sites near Cu^{2+} of *An*LPMO15g; yellow dots are mutation sites for *An*LPMO15g; L1, L2, L3 and L4 were Loop rings near the active center)

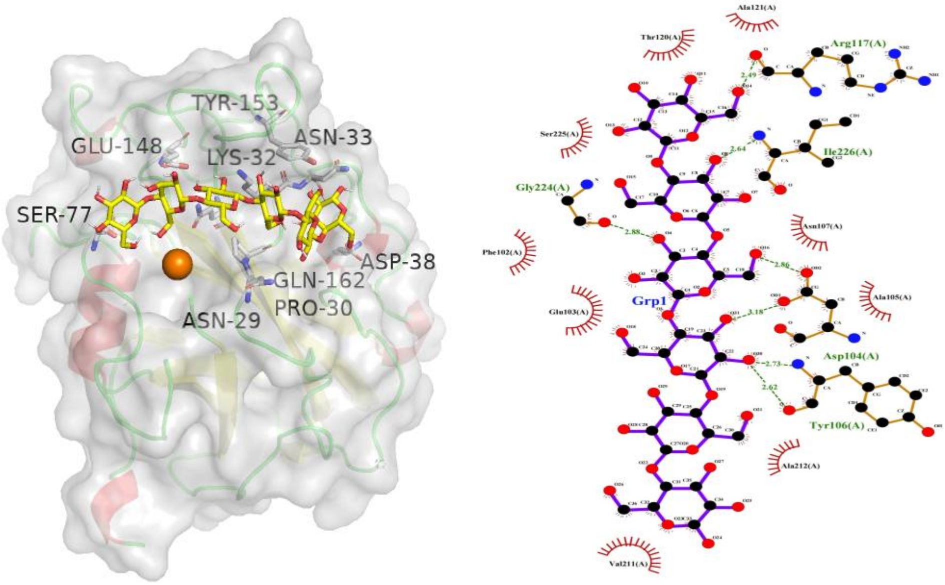

(a) (b)

Figure S2. Optimal conformation diagram of *An*LPMOI5g docking with cellohexose.

(a) the overall picture; (b) 2D schematic diagram

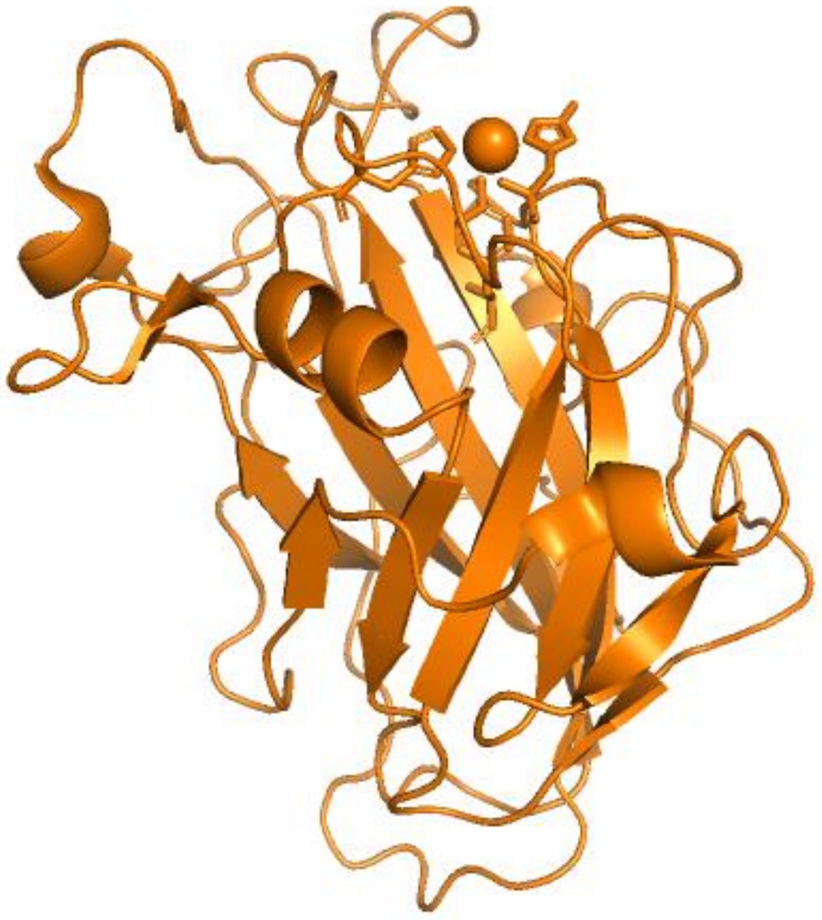

Figure S3. The three-dimensional structure of *An*LPMO15g

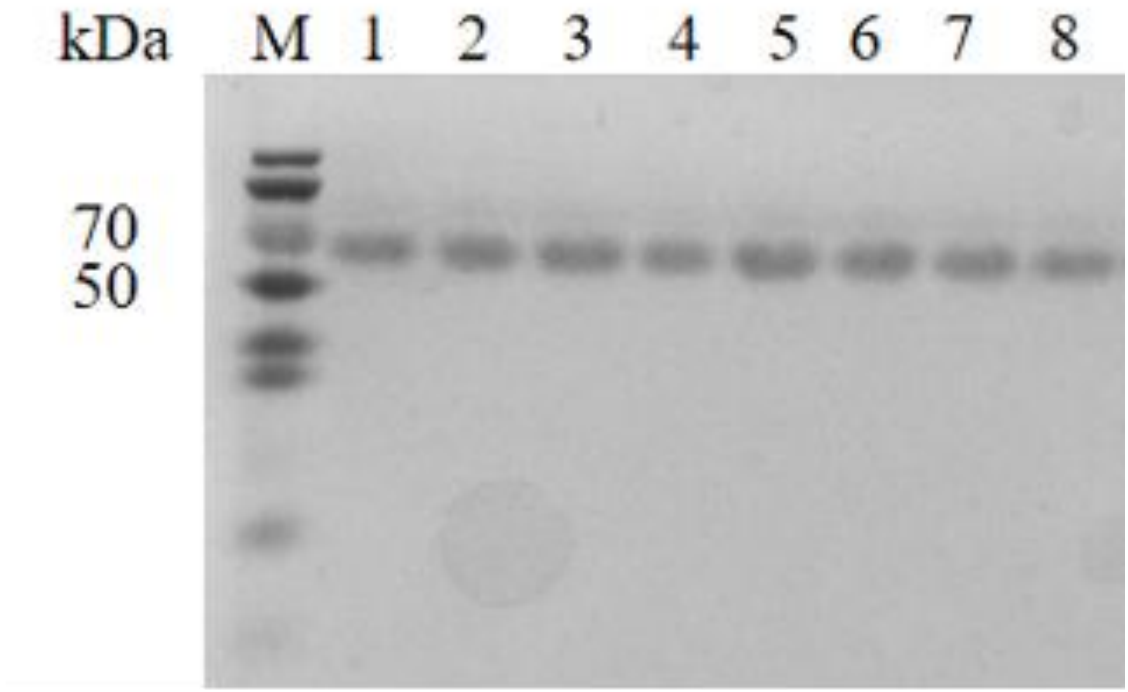

Figure S4. SDS-PAGE results of recombinant *Pichia pastoris*. M: Protein Marker, lanes 1, 2, 3, 4, 5, 6, 7, 8 represent recombinant proteins S197H, S197F, E185V, E185L, E185M, E185I, Q108M, and A249P, respectively. No editing was applied beyond standard brightness/contrast adjustment for the whole image.

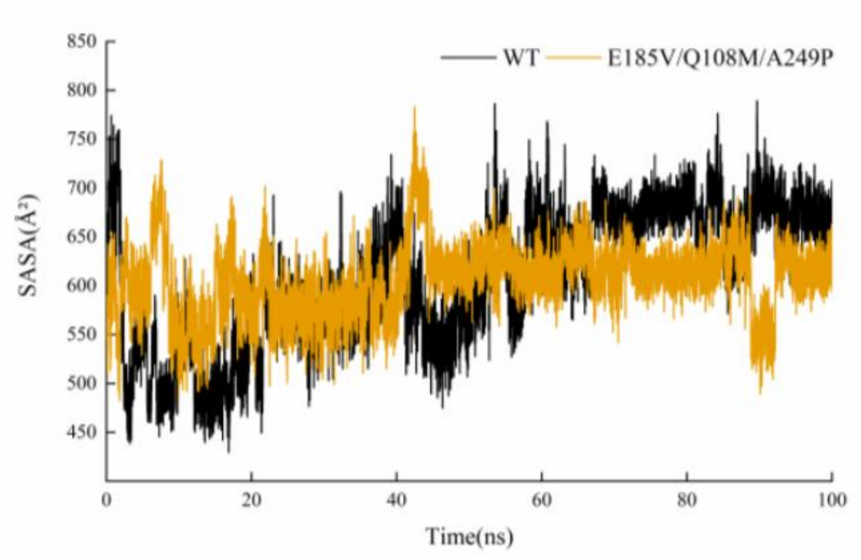

Figure S5. Solvent accessible surface area (SASA) analysis of E185V/Q108M/A249P
